# Editorial: Metastatic hormone-sensitive prostate cancer: new therapeutic strategies to improve patient outcome

**DOI:** 10.3389/fonc.2025.1589694

**Published:** 2025-04-09

**Authors:** Ludmilla T. D. Chinen, Marcus V. Sadi

**Affiliations:** ^1^ Hcor, Associação Beneficente Siria, São Paulo, Brazil; ^2^ Hospital Amaral Carvalho, Jau, São Paulo, Brazil; ^3^ Federal University of São Paulo, São Paulo, Brazil

**Keywords:** advanced prostate cancer, antiandrogens, ARSI, hormone blockage, prognosis

Advanced prostate cancer is a heterogeneous disease presenting complex challenges in clinical management. The four studies reviewed in this editorial contribute with valuable insights into optimizing treatment strategies, improving patient outcomes, and understanding the broader impacts of therapy on quality of life.

Androgen deprivation therapy (ADT) is the cornerstone of treatment for men with advanced prostate cancer. Monotherapy with ADT is no longer considered the standard of care for the initial treatment for metastatic hormone-sensitive prostate cancer (mHSPC) and the association of ADT plus the use of one the newer androgen receptor signaling inhibitor pathway (ARSI) is currently recommended. In special higher risk cases triple therapy which includes the addition of docetaxel (DO) is in vogue, although there is insufficient evidence to use triple therapy to treat all mHSPC patients.

But the use of the combination therapy may be restricted due to reimbursement limitations by different public health insurance systems and many patients still receive ADT monotherapy or a combination of ADT and bicalutamide, an older selective androgen receptor antagonist that falls behind the newer ARSI in terms of progression free survival (PFS).

In a recent systematic review of 29 studies covering North America, Europe and Asia ADT monotherapy was predominantly used across all geographies. ADT + ARPI was used in 20–40% of the patients, followed by ADT + DOC (10–20%). In Asia, each combination remained very low. Abiraterone was the most frequently used ARPI, followed by enzalutamide (1).

One of the major clinical issues regarding the use of ADT therapy in mHSPC relates to cardiovascular toxicity. The extent to which ADT may contribute to excess cardiovascular events remains controversial, but it is even more importantly today due to the use of ADT with ARSIs.

The paper: “*Mitigating Cardiovascular Toxicity in Prostate Cancer Treatment*” highlights the cardiovascular risks of current therapies and suggests de-escalation treatment strategies for patients with favorable responses. The use of biomarkers such as AR-V7 to guide treatment decisions and interventions to optimize cardiovascular health such as exercise and managing modifiable risk factors is proposed. Also, authors cite a score published by Bathia et al. (2), which suggest an easy ABCDE algorithm to be recommended as follows:

“A”: increasing awareness of cardiovascular signs and symptoms plus daily;
“B”: blood pressure in upper limits of 140/90mmHg (revision of these values may be in order);
“C”: statin therapy for pre-existing hyperlipidemia and smoking cessation;
“D”: good control of diabetes and correction of diet;
“E”: exercise.

Other two papers in this Research Topic relate to treatment options in advanced prostate cancer.

“*Clinical Experience with Rezvilutamide in Combination with ADT*” explores the use of rezvilutamide, a new androgen receptor (AR) antagonist, in combination with ADT for mHSPC and locally advanced prostate cancer. It is a series of 4 case reports with promising early results, including significant declines in PSA levels and improvements in radiographic progression. However, the small cohort and limited follow-up underscores the need for larger studies to validate these findings.

The paper “*Comparing ARSIs for mHSPC Treatment: Enzalutamide, Apalutamide, and Abiraterone*”, is a retrospective study that compares four selective androgen receptor antagonists: older bicalutamide, abiraterone and two newer ARSIs – enzalutamide and apalutamide, in combination with ADT for mHSPC. Their findings demonstrate that enzalutamide and apalutamide outperform bicalutamide in delaying disease progression, with enzalutamide showing significant benefits across multiple survival metrics, including PSA-PFS, radiographic PFS, and overall survival (OS). Abiraterone did not demonstrate any significant advantages over the other ARSIs. The analysis of PSA kinetics also revealed that enzalutamide and apalutamide achieved faster and more pronounced responses, contributing to their preferred position in the treatment hierarchy. While safety profiles seemed similar across the drugs, the study reinforces the need for careful selection of therapy based on individual patient factors.

Despite the significant improvements in survival with the new combination therapies for mHSPC, most patients eventually develop biochemical and/or clinical progression with low levels of testosterone known as castration-resistant prostate cancer (CRPC).

The majority of CRPC still remain dependent on AR signaling. There are several important factors that may contribute to AR reactivation despite castrate serum levels of androgens. These include changes in AR expression and structure through gene amplification, mutations, alternative splicing, cell signaling changes and coregulator proteins among others mechanisms. However, the introduction of more effective AR-targeted therapies such as enzalutamide, apalutamide and darolutamide have caused an increase of AR low/negative CRPC patients (3). This is clinically significant because there are fewer options for treatment of AR-independent CRPC and real-life responses to treatment are not very predictable with current biomarkers.

In the paper “*Combined HALP and TTCR Scores for Prognostication in mCRPC*”, the authors explore the importance of inflammation on advanced prostate cancer (4). In the study the predictive value of combining the HALP (hemoglobin, albumin, lymphocyte, and platelet) ratio scores with time to castration resistance (TTCR) was evaluated to estimate OS and PFS. Their results emphasize the importance of combining these parameters as a prognostic tool to risk stratify patients. Patients with low HALP scores and shorter TTCR had significantly worse survival outcomes. Besides its retrospective design and the need for further external validation, this paper shows that useful simple and cheap hematological parameters may provide important CRPC prognosis by analyzing the inflammatory status of these patients.

In conclusion, these studies represent advances in the management of prostate cancer, shedding light on important areas such as prognostication, cardiovascular risk management and the comparative efficacy of different ARSIs. Together, they reflect an evolving understanding of how to balance the effectiveness of therapies with patient safety and quality of life. They also highlight the need for larger, prospective trials to confirm these findings and to further refine treatment strategies for advanced prostate cancer.
